# Structural and kinetic characterization of an acetoacetyl-Coenzyme A: acetate Coenzyme A transferase from the extreme thermophile *Thermosipho melanesiensis*

**DOI:** 10.1042/BCJ20240747

**Published:** 2025-02-18

**Authors:** Ryan G. Bing, Greg K. Buhrman, Kathryne C. Ford, Christopher T. Straub, Tunyaboon Laemthong, Robert B. Rose, Michael W.W. Adams, Robert M. Kelly

**Affiliations:** 1 Department of Chemical & Biomolecular Engineering, North Carolina State University, Raleigh, NC 27695, U.S.A.; 2 Biomanufacturing Training & Education Center, North Carolina State University, Raleigh, NC 27695, U.S.A.; 3 Department of Molecular & Structural Biochemistry, North Carolina State University, Raleigh, NC 27695, U.S.A.; 4 Department of Biochemistry and Molecular Biology, University of Georgia, Athens, GA 30602, U.S.A.

**Keywords:** acetone biosynthesis, Coenzyme A transferase, thermophile

## Abstract

Family 1 Coenzyme A transferases (CtfAB) from the extremely thermophilic bacterium, *Thermosipho melanesiensis*, has been used for *in vivo* acetone production up to 70°C. This enzyme has tentatively been identified as the rate-limiting step, due to its relatively low-binding affinity for acetate. However, existing kinetic and mechanistic studies on this enzyme are insufficient to evaluate this hypothesis. Here, kinetic analysis of purified recombinant *T. melanesiensis* CtfAB showed that it has a ping-pong bi-bi mechanism typical of Coenzyme A (CoA) transferases with K_m_ values for acetate and acetoacetyl-CoA of 85 mM and 135 μM, respectively. Product inhibition by acetyl-CoA was competitive with respect to acetoacetyl-CoA and non-competitive with respect to acetate. Crystal structures of wild-type and mutant *T. melanesiensis* CtfAB were solved in the presence of acetate and in the presence or absence of acetyl-CoA. These structures led to a proposed structural basis for the competitive and non-competitive inhibition of acetyl-CoA: acetate binds independently of acetyl-CoA in an apparent low-affinity binding pocket in CtfA that is directly adjacent to a catalytic glutamate in CtfB. Similar to other CoA transferases, acetyl-CoA is bound in an apparent high-affinity binding site in CtfB with most interactions occurring between the phospho-ADP of CoA and CtfB residues far from the acetate binding pocket. This structural-based mechanism also explains the organic acid promiscuity of CtfAB. High-affinity interactions are predominantly between the conserved phospho-ADP of CoA, and the variable organic acid binding site is a low-affinity binding site with few specific interactions.

## Introduction

Fermentative production of acetone has been of interest for over a century. The bulk of this work has its roots in acetone-butanol-ethanol (ABE) fermentation [1,2], the industrialization of which played a critical role during World War I and employed the mesophile, *Clostridium acetobutylicum*, a bacterium isolated by Chaim Weizmann [3]. In the mid-1900s, ABE fermentation was displaced by petroleum-derived chemicals. However, more recently, there has been renewed interest in ABE fermentation, particularly for acetone and butanol production, as the world searches for sustainable and renewable alternatives to fossil-derived products. Much of the recent work in microbiological solvent production has focused on improvements in mesophilic bacteria, ranging from native ABE Clostridia (*C. acetobutylicum* and *Clostridium beijerinckii*) [4] to non-native ABE bacteria (such as *Clostridium autoethanogenum*) [5,6] and *Escherichia coli* [7]. Thermophilic production of acetone could have advantages over mesophilic alternatives by leveraging higher temperatures and the volatility of acetone for novel separation techniques and contamination resistance [8,9]. Production of acetone in thermophilic bacteria has been demonstrated in *Anaerocellum (f. Caldicellulosiruptor) bescii* [9,10] and *Moorella thermoacetica* [11] using a set of enzymes previously identified for thermostable acetone production [9].

Native ABE fermentation proceeds in two phases: acidogenesis, where acetate and butyrate are accumulated concomitant with a pH drop, then a shift to solventogenesis induced by the low pH where the organic acids are converted into ABE. Evolution of solventogenesis under low pH allows cells to outcompete non-solvent producers by minimizing further acidification. ABE are produced in ratios that allow electron carriers to be redox balanced (i.e. NADH, NADPH, and reduced ferredoxin). ABE fermentation relies on family 1 Coenzyme A transferases (CtfAB) to recycle acetate and butyrate and produce solvent intermediates. Efforts have been conducted to separate ABE fermentation from one another, or alternatively engineer a single solvent-producing pathway into non-native ABE bacteria. While much of this work has focused on the butanol production [12,13], acetone production is also of interest.

**Scheme 1:**
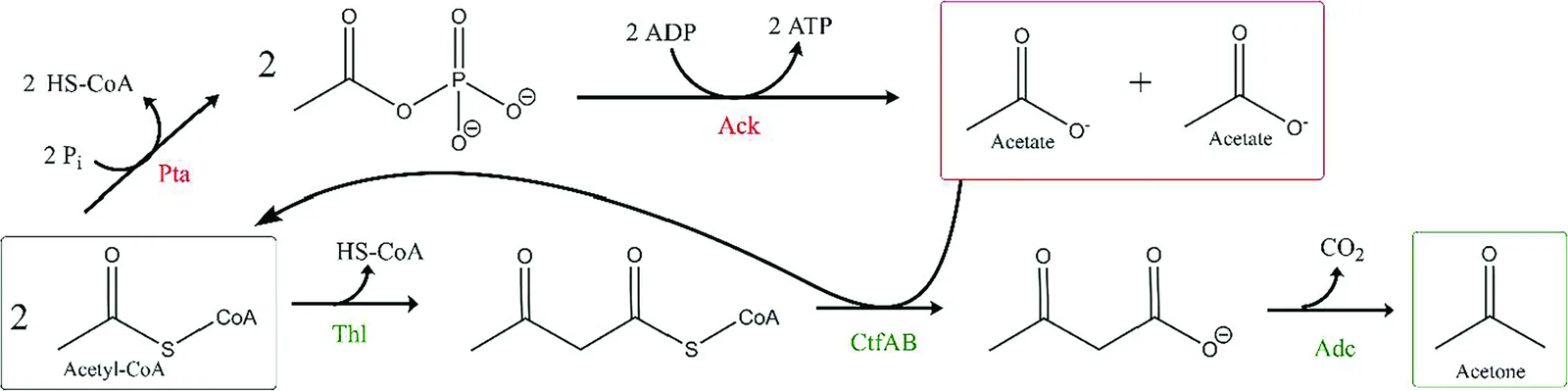
Metabolic path to acetone and acetate from acetyl-CoA. Pta = acetyl-CoA:phosphate acetyltransferase; Ack = acetate kinase; Thl = thiolase; CtfAB = acetoacetyl-Coenzyme A: acetate Coenzyme A transferase; Adc = acetoacetate decarboxylase.

The acetone formation pathway (**Scheme 1**) has two branches. The first is acetate formation (acidogenesis) that produces ATP and is favored by native ABE fermenters when pH is high enough because of the bioenergetic advantage. The second branch produces acetone via three enzymes. Thiolase (Thl) condenses two acetyl-CoA molecules to form acetoacetyl-CoA and CoASH. CtfAB then transfers the CoA from acetoacetyl-CoA to acetate to reform an acetyl-CoA and produce acetoacetate. Lastly, acetoacetate is converted into acetone and CO_2_ by acetoacetate decarboxylase (Adc). The organic acid specificity of CtfAB is typically relatively promiscuous, since native ABE bacteria utilize CtfAB to recycle both acetate (to acetyl-CoA) and butyrate (to butyryl-CoA). Currently, only mesophiles have been identified as native acetone producers. However, homologs of Thl and CtfAB were identified in thermophiles and were combined with the highly thermostable Adc from *C. acetobutylicum* to produce acetone at elevated temperatures in a synthetic pathway *in vitro* [9].

Work on mesophilic and thermophilic acetone production has connected increased acetone production with increased expression of CtfAB [10,14]. Additionally, for mesophilic acetone production, specific CtfAB variants produced significant gains in acetone production. The ‘best’ CtfAB identified to date is from *C. beijerinckii* strain DJ033 [14]. Taken together, this implicates CtfAB as a critical rate-limiting step in the acetone production pathway. Michaelis coefficients (K_m_) for organic acids, particularly acetate, in the mM range have been reported for CtfABs [9]. This reflects the high organic acid concentrations and lower pHs in native ABE fermentation needed for acetone formation. As a result, efforts to engineer homoacetone fermentation still need acetate, requiring up to 50 mM exogenous acetate added to maximize non-native acetone production [10]. Currently, only one thermophilic CtfAB, from *Thermosipho melanesiensis*, has been characterized [9]. However, this previous work did not report detailed kinetic parameters of *T. melansiensis* CtfAB and was impacted by the instability of the beta subunit due to purification of subunits individually [9]. Precise kinetic parameters and mechanistic insights of CtfABs are needed to understand what makes CtfAB a rate-limiting step in acetone production, and what underlies the mM level organic acid K_m_ values; these are presumably the reason why high levels of acetate are needed to drive acetone production *in vivo*.

If acetone can be produced at higher temperatures through metabolic engineering of thermophilic microorganisms, the volatility of this solvent can be exploited to simplify its recovery from fermentation broths through bioreactive distillation [9,10,15]. If acetone is formed at high enough concentrations, it can be flash-separated and recovered through condensation. An efficient thermophilic CtfAB that requires less accumulation of acetate is clearly critical to this strategy, although such an enzyme has not yet been identified. The goal here was to characterize the thermophilic CtfAB from *T. melanesiensis* to determine how its kinetic and structural properties compare with those of its mesophilic counterparts. This information provides the foundation to improve this enzyme for thermophilic bacterial acetone production.

## Results and discussion

### Expression and purification of CtfAB

**Figure 1:**
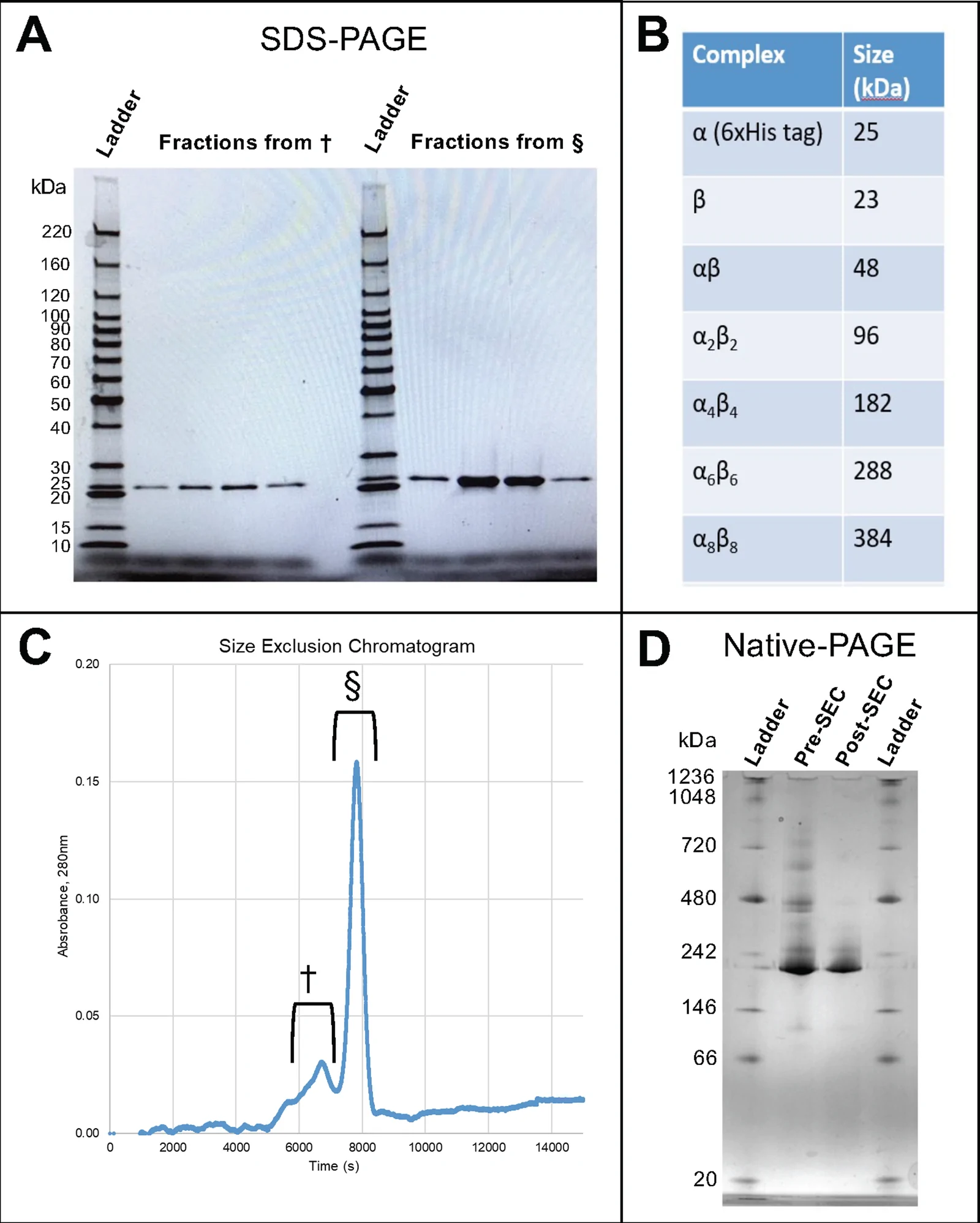
*T. melanesiensis* CtfAB purification, molecular mass, and oligomerization. **[**A**]** SDS-PAGE gel of FPLC fractions from size exclusion chromatography (SEC). **[**B**]** Table of Ctf subunit and oligomer expected molecular masses. **[**C**]** Chromatogram from size exclusion purification; 280 nm absorbance versus elution time (**S**). Chromatogram regions † and § correspond to fractions in **[**A**]**, as indicated. **[**D**]** Native-PAGE of pooled CtfAB sample after nickel affinity chromatography (pre-SEC) and after size exclusion chromatography (post-SEC).

Previous characterization of the thermophilic CtfAB from *T. melanesiensis* did not report detailed kinetic parameters and struggled with instability of the beta subunit due to the purification method. Here, the genes encoding the two subunits of *T. mel* CtfAB were co-expressed in *E. coli* with an N-terminal 6x histidine tag on the A subunit. CtfAB was purified by heat treatment (65°C for 30 min) followed by nickel affinity and size exclusion chromatography. This approach contrasts with the previous work with *T. mel* CtfAB where the individual subunits were recombinantly expressed separately [9,10]. Expression and purification of the holoenzyme improved the stability of the beta subunit over separate purifications of alpha (CtfA) and beta (CtfB) subunits, where CtfB had stability issues when expressed alone [9]. SDS-PAGE analysis indicated that the resulting holoenzyme was highly purified (Figure 1A) and the expected molecular subunit masses were observed (M_r_ 23–25 kDa (Figure 1A and B). The size exclusion chromatogram displayed multiple major peaks (Figure 1C); the analysis of two of these major peaks (†, §) by SDS-PAGE showed that both subunits were present (Figure 1A). This indicated multiple oligomer states of the CtfAB complex exist. Native-PAGE analysis of pooled Fast protein liquid chromatography (FPLC) fractions of *T. melanesiensis* CtfAB after nickel affinity chromatography (pre-SEC) and after SEC (post-SEC) (Figure 1D) confirmed the presence of multiple oligomeric forms of CtfAB in pre-SEC and the ability of SEC to purify the predominate oligomeric form (post-SEC Region §). Region † represents the higher molecular mass oligomers of CtfAB, which can be seen on the native-PAGE in Figure 1D. Fractions corresponding to Region § were pooled to give the purified CtfAB enzyme that was used for kinetic and crystallographic analyses. Distributions of oligomeric forms were observed to change in different buffer compositions (i.e. chromatography buffers compared with crystallization buffer, where less high molecular weight oligomers were observed in the crystallization buffer, and reversion to the oligomer distribution shown in Figure 1D ‘pre-SEC’ was observed when the ‘post-SEC’ fraction was stored at room temperature for >4 h prior to native-PAGE), which leaves the *in vivo* oligomeric distribution unknown.

Because multiple oligomeric states of CtfAB were found during protein purification, an effort was made to ascertain whether or not higher order crystallographic symmetry oligomers were likely to be biologically significant using Evolutionary Protein-Protein Interface Classifier (EPPIC). EPPIC is a web-based bioinformatics server that predicts the quaternary structure of protein crystals using an evolutionary-based algorithm that scores protein interfaces in terms of confidence values that a given interface is biologically significant [16]. An EPPIC analysis of CtfAB found that the protein interfaces which make up the CtfAB heterodimer interface and the CtfAB–CtfAB heterotetramer interfaces are highly predicted to have biological significance as opposed to the crystallographic octamer interfaces which are highly predicted to be crystal lattice contacts that do not have biological significance (Supplementary Figure S1, Figure S2). While it is clear from SEC and native-PAGE that multiple higher order oligomeric states of CtfAB do exist in solution, the heterotetramer is the highest-ordered state apparent in CtfAB crystals.

#### *T. melanesiensis* CtfAB mutations

In addition to the several wild-type (WT) *T. mel* CtfAB (WT-CtfAB), several mutant enzymes were produced: CtfB (beta subunit) [E46D], [E46A], [E46S], [I25K], [Q105A], [Q105E], [Q105E, G103A], [F42T, Q44E], [F42T, S45C]; CtfA (alpha subunit) [L25M, F54L, T78L], [P118E]. Mutations were selected based on available and predicted structures and mechanism insights for CoA-transferase active sites and substrate-binding pockets, where most of the residues tested were in proximity to the catalytic residue (E46) or the CoA and organic acid binding pockets. These are discussed in more detail in later sections in context of protein structure. In addition, CtfAB from *C. beijerinckii* strain DJ033 (T_opt_~30°C) and the chimeric *T. mel* CtfA–DJ033 CtfB were purified. These mutants had three purposes: to improve functionality in the *in vitro* acetone pathway at 70°C, create catalytically dead *T. mel* CtfAB (E46A and E46S), and to compare against a reportedly highly functional mesophilic CtfAB (DJ033). These enzymes were tested in the *in vitro* acetone pathway which confirmed that E46A and E46S had no activity. Based on previous reports [17–19], mutation of the catalytic residue on the beta subunit (residue 46) from a glutamate to aspartate (βE46D) should convert the CoA transferase into a CoA hydrolase. These reports include a patent that claims that this mutation, at least in some enzymes, should have preferential specificity for acetoacetyl-CoA over acetyl-CoA by at least 10-fold [18]. Successful conversion of *T. mel* CtfAB into a CoA hydrolase with high specificity for acetoacetyl-CoA over acetyl-CoA would allow *in vivo* acetone pathways to avoid the need to accumulate acetate. Here, creation of the E46D mutant in the B subunit converted the transferase into a hydrolase, with activity confirmed on acetoacetyl-CoA. Evaluation using the *in vitro* acetone pathway assay at 70°C showed that, in contrast with the WT enzyme, activity of the E46D mutant did not require acetate; acetone formation was the same at 150 mM or 0 mM potassium acetate (0.2–0.3 mM produced from 5 mM acetyl-CoA). The WT enzyme produced 4.2 mM and 0.1 mM acetone, respectively, under these conditions. This is most likely due to non-specific hydrolysis of acyl-CoA molecules, where acetyl-CoA is converted into CoASH and acetate, significantly reducing available acetyl-CoA that can be converted into acetoacetyl-CoA. The other mutations were tested in the *in vitro* acetone pathway at these conditions and their activities in comparison with the WT enzyme were as follows: I25K – no effect; βE46A – no activity; βE46S – no activity; α[T78L, L25M, F54L] – no effect; αP118E – no effect; βQ105E – no effect; βQ105A – no effect; β[Q105E,G103A] – no effect; β[Q44E, F42T] – no effect; β[S45C, F42T] – no effect; β[replaced with DJ033 subunit] – 20% activity at 70°C; Mesophilic (~30°C optimum) DJ033 CtfAB – < 5% activity at 70°C. Lower activity of DJ033 WT and chimeric enzymes at 70°C is likely due to decreased thermostability.

## Kinetics (ping-pong bi-bi) of *T. melanesiensis* CtfAB

CtfAB follow ping-pong bi-bi kinetics [9,20,21]. Here, the detailed kinetic parameters of the *T. mel* CtfAB were determined within the context of the acetone production pathway (**Scheme 1**), where the forward reaction is in the acetoacetate/acetyl-CoA formation direction and the reverse reaction is in the acetoacetyl-CoA/acetate formation direction. **Equation 1** summarizes the reaction: A = acetoacetyl-CoA, B = acetate, *P* = acetoacetate, Q = acetyl-CoA:


(1) A+B↔P+Q

Briefly, the mechanism proceeds by binding acetoacetyl-CoA (A). The CoA is then transferred to the enzyme catalytic site (β, E46), after which acetoacetate (P) is released. Acetate (B) binds and links to the CoA, which is then released from the enzyme as acetyl-CoA (Q). In the context of the acetone pathway, the acetoacetate (P) formed is rapidly converted by the Adc into acetone and CO_2_ [9]. As such, a ping-pong bi-bi rate equation with inhibition by the presence of Q (acetyl-CoA) was fitted for the forward reaction (**Equation 2**) [21].


(2) $$\nu_{fwd}={\left[\frac{1}{V_{fwd}}+\frac{K_{m,A}}{V_{fwd}\left[A\right]}\left(1+\frac{\left[Q\right]}{K_{i,Q}}\right)+\frac{K_{m,B}}{V_{fwd}\left[B\right]}+\frac{K_{i,B}K_{m,A}\left[Q\right]}{V_{fwd}K_{i,Q}\left[A\right]\left[B\right]}\right]}^{-1}$$

Additionally, the reverse reaction rate without inhibition parameters was fitted as follows:


(3) $$\nu_{rev}=\frac{V_{rev}\left[P\right]\left[Q\right]}{K_{m,Q}\left[P\right]+K_{m,P}\left[Q\right]+\left[P\right]\left[Q\right]}$$

**Figure 2:**
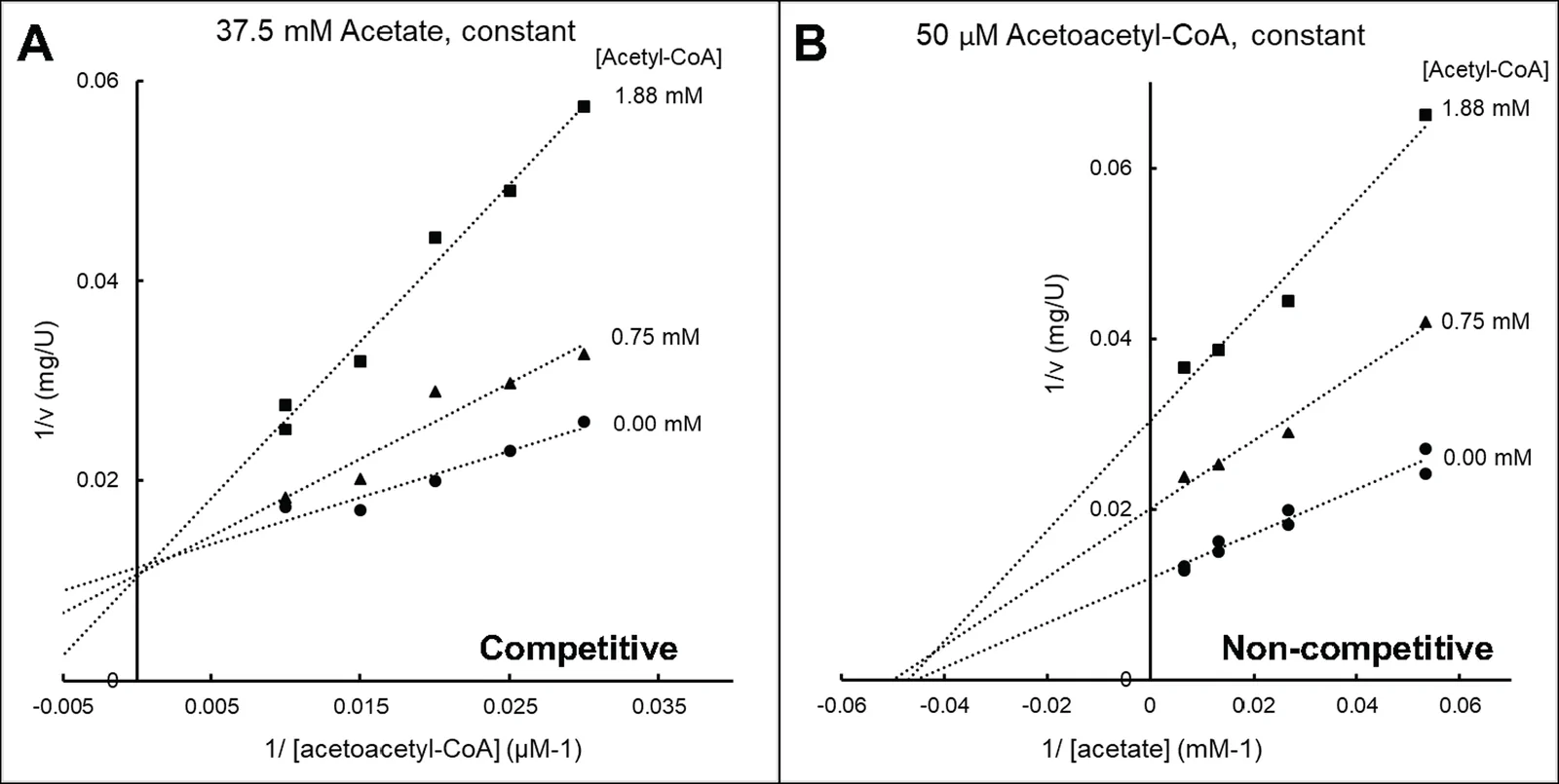
Inhibition by acetyl-CoA on CtfAB forward reaction. **[**A**]** Double-inverse plot of reaction rate (ν_fwd_) for variable acetoacetyl-CoA and acetyl-CoA concentrations for constant acetate at 37.5 mM. **[**B**]** Double-inverse plot of reaction rate (ν_fwd_) for variable acetate and acetyl-CoA concentrations for constant acetoacetyl-CoA at 50 µM.

Table 1 summarizes the estimated parameters. Notably, the K_m_ for acetate is relatively high (85 ± 16 mM), which is 2–3 orders of magnitude larger than the CoA-associated K_m_ values and 1–2 orders of magnitude larger than the K_m_ value for acetoacetate. The maximum reaction velocity of the reverse reaction was ~1/6 of the forward reaction. Forward reaction inhibition by acetyl-CoA is significant (Figure 2) and competitive with respect to acetoacetyl-CoA and non-competitive with respect to acetate. The high K_m_ for acetate reflects the conditions where native ABE bacteria produce acetone after accumulating high concentrations of organic acids. Inhibition by acetyl-CoA is also overcome by high levels of acetate. This correlates with the need to add exogenous acetate to drive *in vivo* acetone production in *Anaerocellum (f. Caldicellulosiruptor) bescii* [10].

**Table 1:** Ping-pong bi-bi kinetics with inhibition for *T. melanesiensis* CtfAB

Forward reaction rate equation (ν_fwd_), *R^2^* = 0.9433, *n* = 66 ± std error		
V_fwd_	320 ± 48	U/mg-CtfAB
K_m,A_	138 ± 28.8	µm
K_m,B_	8.47 × 10^4^ ± 1.56 x10^4^	µm
K_i,B_	2.47 × 10^4^ ± 1.66 x10^4^	µm
K_i,Q_	1267 ± 276	µm
**Reverse reaction rate equation (ν_rev_), *R^2^* = 0.9787, *n* = 24 ± std error**		
V_rev_	−54.1 ± 4.1	U/mg-CtfAB
K_m,Q_	322 ± 41	µm
K_m,P_	1655 ± 331	µm
U = µmol-acetoacetyl-CoA-consumed/min		

While the mechanism of CtfAB is well established, the kinetic parameters of specific CoA transferases are not widely reported. Available literature is limited regarding the detailed kinetics for acetoacetyl-CoA:acetate CoA transferase (EC 2.8.3.8). The CtfAB from *Clostridium acetobutylicum,* an OXCT1 family member with no structural information available*,* has a high K_m_ for acetate and lower K_m_ for CoA. However, detailed fitting of the ping-pong bi-bi kinetics was not done, and inhibition parameters were only reported with butyrate not acetate [22]. Notably, the *C. acetobutylum* CtfAB K_m_ for acetate is 1,200 mM (compared with 85 mM for *T. mel* CtfAB), but it shares a similar K_m_ for acetoacetyl-CoA (21 vs 138 µM, *C. acetobutylicum* vs. *T. melanesiensis*). As such, the data reported here are the most complete acetoacetyl-CoA:acetate CoA transferase kinetic data for both mesophilic and thermophilic enzymes. Kinetics for mesophilic succinyl CoA:acetoacetate CoA transferase from pig heart (EC 2.8.3.5) have been determined and are similar to those of the *T. mel* CtfAB for the acetoacetyl substrates [23]. The pig heart CoA transferase is in similar order of magnitude for V_max_ (V_fwd_ = 716 U/mg for Ctf, V_rev_ = 28.3 U/mg-Ctf) and K_m_ for acetoacetyl-CoA and acetoacetate (720 µM and 200 µM, respectively). Additionally, the K_m_ values for succinate and succinyl-CoA for pig heart CoA transferase (36 mM and 4.2 mM, respectively) are comparable to those measured for acetate and acetyl-CoA with *T. mel* CtfAB, with relatively high K_m_ values for the organic acid. Inhibition coefficients (K_i_) are similar to K_m_ values of their respective substrates, as expected in both cases. The large differences in substrate K_m_ values, especially between organic acids in both cases (acetoacetate compared with either succinate or acetate), raise the question as what is causing these differences. In the context of non-native acetone production, large amounts of acetate are not desirable, so understanding the mechanism of organic acid substrate specificity is needed to help guide efforts to improve the acetone formation pathway.

### Structure and phylogenetic analysis

**Figure 3:**
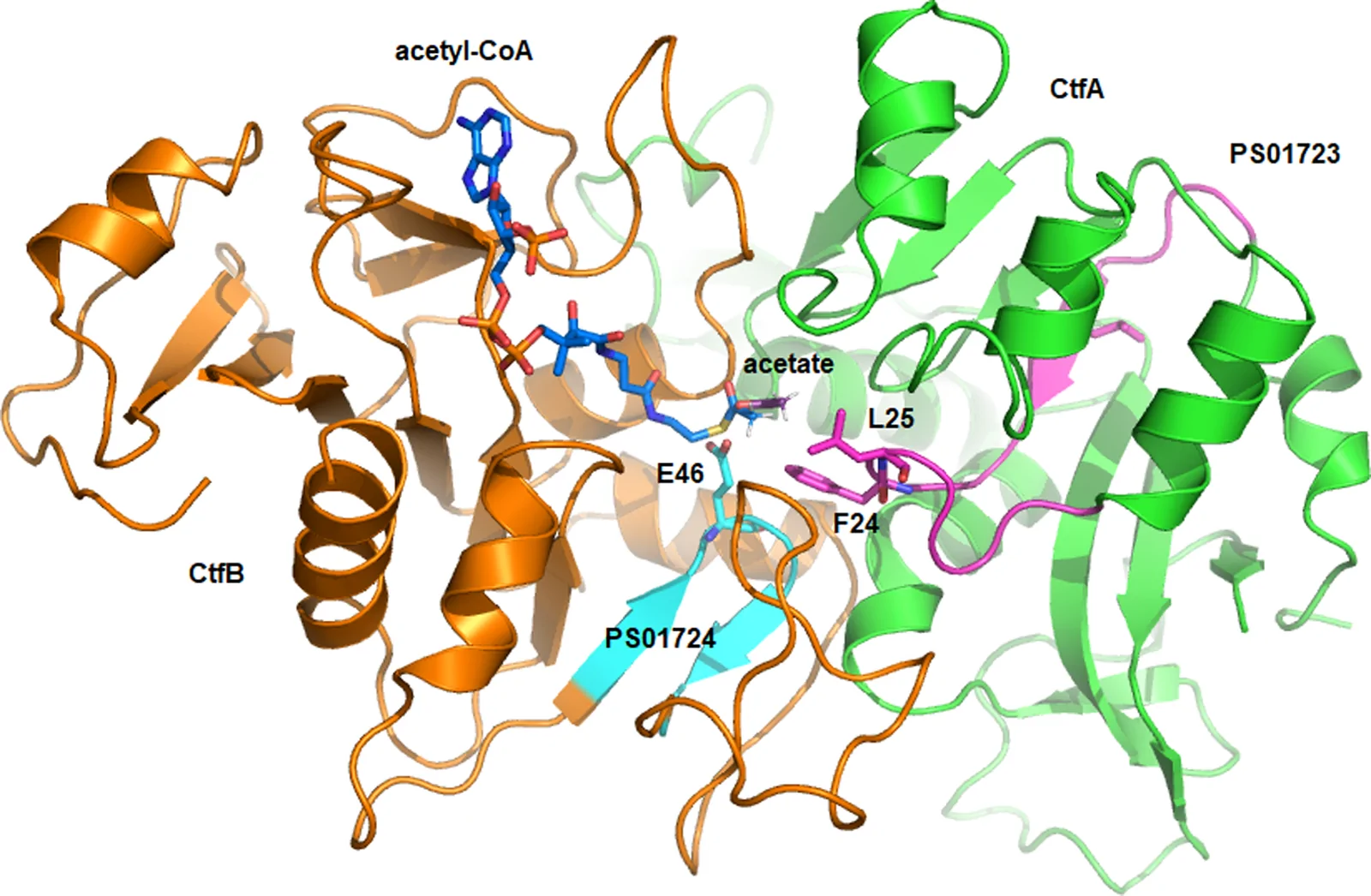
CtfAB heterodimer with PS01723 and PS01724 CoA family signatures. PS01723 is shown in magenta, and PS01724 is shown in cyan. Acetyl-CoA is shown with blue carbon atoms in stick and acetate shown with purple carbon atoms in stick. Figure created in PyMOL.

CoA transferases are divided into three families based on their sequence, structure, and activity. CtfAB is a member of family 1 CoA transferases [9]. Traditionally, CtfAB have been identified based on PROSITE signature sequences, PS01723 and PS01724, found on the A and B subunits, respectively [24]. The structure of the *T. mel.* CtfAB heterodimer bound to acetyl-CoA is shown in Figure 3. CtfB contains the acetyl-CoA binding site and the PS01724 signature sequence which includes the catalytic glutamate residue (E46) sandwiched between two short antiparallel beta strands on the N-terminal end of an eight-stranded mixed beta sheet. This core beta sheet is surrounded by multiple alpha helices, loops, and two short antiparallel beta strands that form a part of the CoA binding site. CtfA contains the acetate binding site and the PS01723 signature sequence. The core of CtfA contains a four-stranded parallel beta sheet flanked by a twisted beta barrel, multiple alpha helices, and connecting loops. The PS01723 signature sequence consists of a loop–beta strand–loop, which places residues L25 and F24 in the acetate-binding pocket at the heterodimer interface. The most similar structural representative to *T. mel* CtfAB in the Protein Data Bank is the *E. coli* acetate CoA-transferase (5DBN). WT-CtfAB and 5DBN superimpose with a Calpha rmsd of 2.5 Å for 842 aligned atoms. The (Supplementary Figure S3) shows a superposition of *T. mel* WT-CtfAB with 5DBN and 3DLX. The core topology of both CtfA and CtfB is structurally well conserved between *T. mel*. CtfAB and *E. coli* acetate CoA-transferase with the exception of an additional short beta strand flanking the PS01724 signature sequence of the *E. coli* acetate CoA-transferase. 3DLX is a human succinyl-CoA:3-oxoacid CoA transferase (SCOT) which transfers CoA from succinyl-CoA to acetoacetate [25]. Mammalian SCOTs are single-chain proteins with an N-terminal domain that is homologous to the alpha subunit of CtfAB and a C-terminal domain that is homologous to the beta subunit of CtfAB. These domains are separated by a flexible linker. WT-CtfAB and 3DLX superimpose with a Calpha rmsd of 1.6 Å for 590 out of 837 aligned residues. The residues which did not align well include the flexible linker, loops at the N and C termini and several surface loops; however, the core of the protein superimposes well.

A recent phylogenetic analysis subdivided CtfAB into three monophyletic families. The Table S1 lists all the OXCT1 family members with deposited PDB structures ranked by sequence identity to *T. mel* CtfAB. In general, CtfB sequence identity is higher than CtfA sequence identity for these OXCT1 family members. Higher sequence conservation in CtfB could be explained by the need to maintain a large, high-affinity binding site for the conserved portion of acyl-CoA substrates, whereas CtfA has less substrate-binding sequence pressure and was allowed to evolve to be more thermostable. The most similar OXCT1 family member is the *E. coli* acetate-CoA transferase (5DBN). The alpha subunits of both proteins are 53% identical by sequence, while the beta subunits are 57% identical by sequence. Both proteins contain the PROSITE signature sequences PS01723 and PS01724. The PSO1723 signature sequence is ((DN)-(GN)**-**x [2]-(LIVMFA) [3]-G-G-F-x [3]-G-x-P). *T. mel* CtfA has the sequence EGATLMIGGFLGVGTP [15–30]. The only deviation of *T. mel* CtfAB from the canonical PS01723 signature is the substitution of E15 for D15. E15 is positioned to hydrogen bond to S43 on an adjacent loop, which may serve to increase the thermal stability of *T. mel.* CtfA. Residues F24 and L25 of the signature sequence form part of a hydrophobic patch in the acetate-binding pocket that is positioned to interact with the methyl group of the co-substrate acetate. F24 is a strictly conserved part of the signature sequence, whereas L25 is variable and is likely dependent on the identity of the substrate. *E. coli* acetate-CoA transferase (5DBN) has the PS01723 signature sequence DGMTIMVGGFMGIGTP with no deviations from the canonical signature sequence. 5DBN has a methionine residue at the position of *T. mel* L25, which maintains the hydrophobic character of the acetate-binding pocket in *E. coli* acetate-CoA transferase, suggesting that acetate binding may be conserved as well. 3DLX is a human OXCT1 family member with 41% sequence identity for the alpha subunit and 52% sequence identity for the beta subunit. In the PS01723 signature sequence, 3DLX has G65 in place of L25. This allows the sidechain of K368 (A70 in *T. mel* CtfA) to form a salt bridge with E118 (T78 in *T. mel* CtfA). In *T. mel* CtfA, there is a contiguous hydrophobic patch between F24, L25, the methyl group of T78, and F54. This hydrophobic patch could provide both binding affinity for acetate and improved thermal stability. This hydrophobic patch is disrupted in 3DLX by the salt bridge between K368 and E118. This may be more favorable for binding the carboxyl group of the 3DLX substrate succinyl-CoA as opposed to the methyl groups of acetoacetyl-CoA and acetate.

#### Acetate-binding site

**Figure 4:**
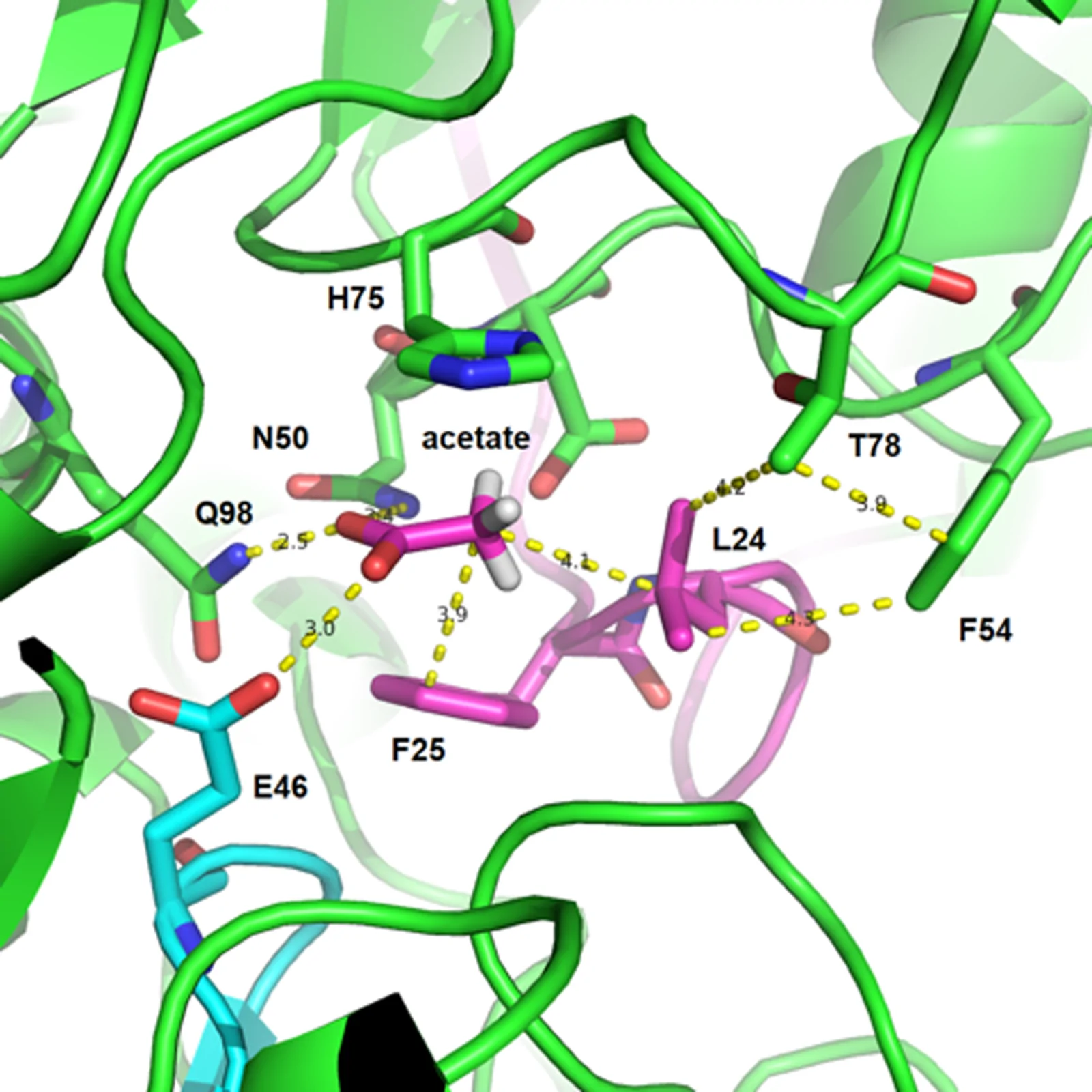
CtfAB acetate binding site. PS01723 signature in magenta, PS017234 signature in cyan. Distances of dashed yellow lines are in Å.

To investigate how acetate binds to *T. mel.* CtfAB, the enzyme was crystallized in a buffer containing 100 mM sodium acetate. Acetate molecules were identified in every CtfAB structure in the substrate-binding pocket directly adjacent to catalytic residue E46. The (Supplementary Figure S4) shows difference density for two acetate molecules in the CtfAB F42TS45C-CoA structure. Acetate 1, colored magenta, is the only location where acetate is bound in the same conformation in each structure. The conserved acetate-binding site in Wt-CtfAB is shown in Figure 4. L25 and F24 of CtfA form a hydrophobic pocket for the methyl group of acetate (301 A). The methyl group of acetate (301 A) is 3.9 Å from the CZ side chain carbon of F24 and 4.0 Å from the CD2 atom of L25. On the opposite side of the binding site, one of the polar oxygens is in good hydrogen bonding distance (2.5 Å) to the side chain NE of CtfA Q98 and 3.4 Å to the side chain ND2 of CtfA N50. The second polar oxygen of acetate is 3.0 Å from the OE2 side chain atom of the catalytic E46 residue. This oxygen is solvent exposed and connected to an extensive water-filled cavity.

Acetate is a substrate that participates in the second half of the ping-pong reaction. In the first half of the CoA transferase reaction (**Eqn. 1**), the catalytic glutamate forms a covalent adduct with CoA, making the glutamate side chain oxygen unavailable for hydrogen bonding with the acetate oxygen. The loss of potential binding interactions with acetate during catalysis could play a role in lowering the substrate-binding affinity during catalysis, thus increasing the K_m_ for acetate. To investigate the large difference in the K_m_ value for acetate between *C. ace* CtfAB and *T. mel* CtfAB, a homology model of *C. ace* CtfAB was superimposed with the *T. mel* CtfAB structure. CtfA sequences are 53% identical and 69% similar, whereas CtfB sequences are 64% identical and 80% similar. The *C .ace* CtfAB homology model has a high global quality estimate score of 0.78 [26]. In the acetate-binding pocket, the core hydrophobic binding pocket residues L25 and F24 are conserved, although *T. mel* CtfA T78 is S80 in *C. ace* CtfA. This results in the loss of a methyl group which forms a part of the hydrophobic pocket in *T. mel* CtfA. Also, *T. mel* CtfA F54 is Y56 in *C. ace* CtfA. F54 forms a part of the extended hydrophobic pocket, 3.9 Å from the T78 methyl group and 4.3 Å from L25. Replacing the hydrophobic F54 with Y56 decreases the overall hydrophobic character of the acetate-binding pocket. Lastly, *T. mel* CtfA H75 is Y77 in C. ace CtfA. H75 is located near the carboxyl group of the acetate molecule and is positioned to provide charge complementarity. Y77 maintains the size and shape of the binding pocket, but it cannot provide charge complementarity for the acetate carboxyl group. Taken together, these amino acid substitutions are consistent with the higher K_m_ value for acetate for *C. ace* CtfAB compared with *T. mel* CtfAB.

##### CoASH-binding site

**Figure 5:**
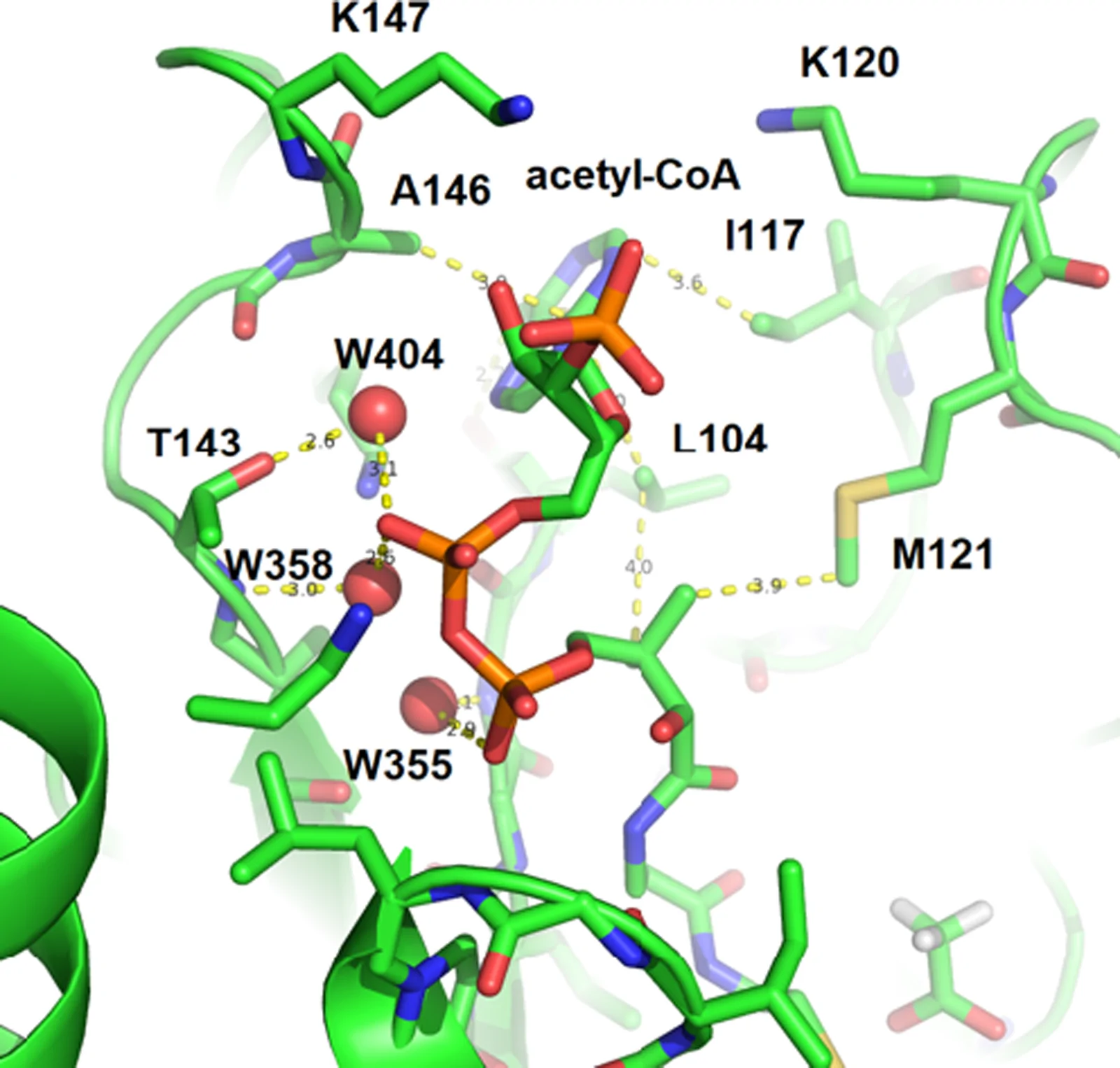
Acetyl-CoA-binding site. The acetyl-CoA phospho-ADP -binding site has multiple water -mediated hydrogen bonds on one face of the binding site and multiple hydrophobic interactions on the opposite face of the binding site. K147 and K120 are positioned to participate in electrostatic interactions with a negatively charged phosphate at the top of the binding site.

The binding site for acetyl-CoA involves primarily CtfB residues that are far from the catalytic glutamate where acetate is bound. For example, E46 CB is 25.5 Å from A146 CB. The adenosine base of acetyl-CoA is wedged between the methyl group of CtfB A146 (chain C) on one side and CtfB L158, I117, and L104 on the other side, shown in Figure 5. M121 extends the hydrophobic patch deeper into the binding site to make hydrophobic contacts with one methyl group of the pantothenic portion of CoA. The second pantothenic methyl group is positioned to make hydrophobic contact with L104. These extensive hydrophobic interactions are likely responsible for the high-binding affinity, but there are also several hydrogen bonds and electrostatic interactions as well. The N6 nitrogen that extends from the adenosine base is 2.6 Å from the main chain carbonyl oxygen of L104. The two phosphates from the ADP tail are hydrogen bonded to the main chain carbonyl oxygen of A151, the main chain amide of T143, and the side chain hydroxyl of T143 via water-mediated hydrogen bonds with water molecules 404, 358, and 355. Water molecules 355 and 358 are low b-factor [20–22], deeply buried water molecules with 4.0 Sigma 2Fo-Fc electron density peaks. Water 404 is partially buried with a corresponding b-factor of 35.7 and a 2 Sigma 2Fo-Fc electron density peak. The acetyl-B-mercaptoethylamine group of acetyl-CoA is modeled only in the heterodimer cleft between chain C and chain D. In the chain A and B heterodimer, only the phosphoadenosine group is included in the model. There is 2Fo-Fc electron density for the modeled acetyl-B-mercaptoethylamine group between chains C and D, but the density includes multiple chain breaks and is weak in places. Interestingly, even with the acetyl-CoA group bound, there are two acetate molecules in the acetate-binding site. Acetate 301D is in the same acetate-binding site previously described for the CtfAB WT structure. Acetate 301C is positioned to h-bond to both acetate 301D and the catalytic Glu46. It seems that the acetate portion of the binding site is flexible enough to accommodate multiple substrates and products, which is consistent with the observed promiscuity of CtfABs for various organic acids (such as *C. ace* CtfAB has activity for acetate, acetoacetate, butyrate, and propionate).

### Active site mutants E46S, E46D

**Figure 6:**
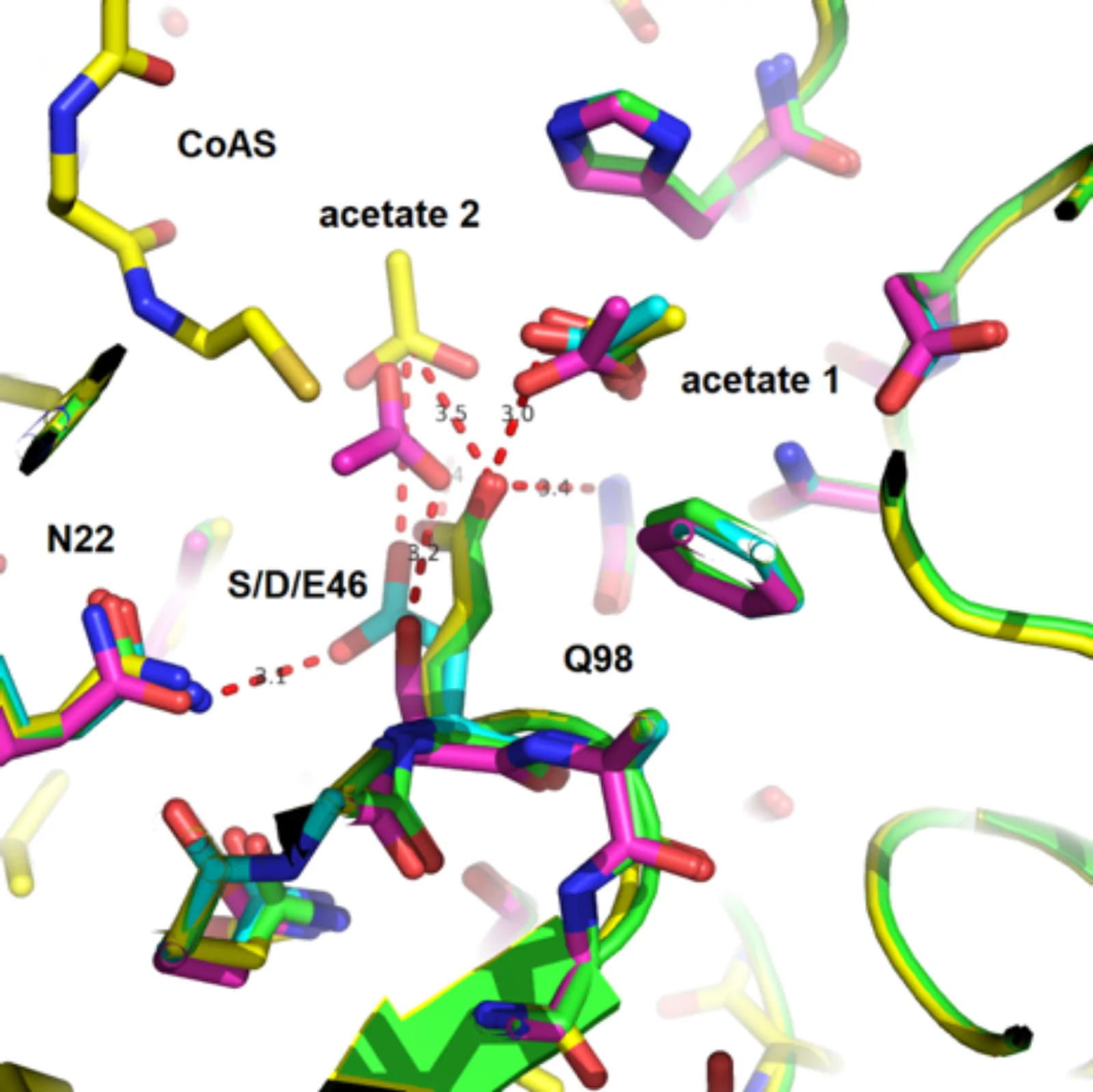
CtfAB E46S and E46D mutant structures. E46S (magenta), E46D (cyan), E46 apo (green), and E46-acetylCoA (yellow) structures were superimposed and figure created in PyMOL.

E46 in the *T. mel* CtfAB beta subunit is a conserved catalytic residue in all CtfAB that form multiple covalent adducts during the reaction. In the first half reaction, it forms an acyl-glutamyl anhydride that is attacked by a CoA thiolate to form a glutamyl-CoA thioester [27]. In other CtfAB, mutation of the catalytic glutamate to aspartate creates an enzyme that is unable to form covalent aspartyl adducts but is competent for hydrolase activity. A proposed mechanism for the hydrolase activity involves the aspartate carboxylate acting as a general base to activate a water molecule for nucleophilic attack [28]. Additional support for the necessity of a carboxylate is that mutation of the catalytic glutamate to serine results in a catalytically inactive enzyme that cannot catalyze either the CoA transferase or hydrolase reaction. The structures and activity assays here of both the E46D and E46S *T. mel.* CtfAB mutants support this mechanism. The E46D mutant is partially active as a hydrolase but cannot catalyze the CoA transferase reaction, as discussed previously. The E46S mutant is catalytically inactive. All of the active site mutants were superimposed with the WT *T. mel.* CtfAB structure and the structure of *T. mel.* CtfAB bound to acetyl-CoA is shown in Figure 6. In these structures, the side chain carboxyl of E46 hydrogen bonds to the side chain nitrogen of Q98 and two acetate molecules

in and around the acetate binding pocket. In contrast, the shorter D46 does not reach into the acetate binding pocket and instead is rotated away from the acetate binding pocket and positioned to form a hydrogen bond with the side chain nitrogen of N22. The E46S mutant hydroxyl group is not within hydrogen bond distance of any protein atoms. Instead, a second acetate molecule binds in the space normally occupied by the catalytic glutamate carboxylate, 3.2 Å from the E46S hydroxyl. Although electron density only supported modeling of CoA-S, two acetate molecules are supported by clear electron density. Acetate 2 is located in between CoA-S and the acetate binding pocket. The carbonyl carbon of Acetate 2 was used as a proxy for the thiol ester carbonyl attacked by either the glutamate carboxylate or the nucleophilic water molecule activated by the aspartate carboxylate. The closest aspartate carboxylate oxygen is 4.1 Å away, and the closest glutamate carboxylate oxygen is 3.5 Å away. The catalytic glutamate is closer, more flexible, and better positioned for direct attack, whereas the aspartate residue is less flexible and partially fixed further away from the active site by the hydrogen bond with N22 making direct attack unlikely, but E46D could activate a water molecule and position it for nucleophilic attack. Based on previous literature, this is the first structural evidence from substrate bound crystal structures that supports the proposed mechanism for how the E46D mutation converts the CoA transferase into a CoA hydrolase ^28^.

## Conclusions

The structural and kinetic characteristics of the CtfAB from the extreme thermophile (growth T_opt_≥70°C) *T. melanesiensis* were investigated to understand how these relate to its possible use in metabolic engineering to exogenously produce acetone at elevated temperatures. Structural insights from substrate bound crystal structures confirm and elaborate on existing literature for CoA transferase mechanisms. Additionally, these insights help explain why binding affinities for organic acids are relatively high in these enzymes. This provides a basis for future work to engineer the organic acid binding pocket for improved specificity. Attempts to convert *T. mel* CtfAB into a hydrolase (β, E46D mutation) worked but was too promiscuous to be useful for specific use in the acetone production pathway. Here, if the specificity of the organic acid binding pocket can be modified in the future, implementation of the E46D mutation should enable acetoacetyl-CoA hydrolase activity without significant activity on acetyl-CoA. If this is accomplished, this would eliminate the need to accumulate significant amounts of acetate *in vivo* for acetone production. The (Supplementary Figure S5) shows sequence alignments with details on specific motifs.

Further, detailed characterization of *T. melanesiensis* CtfAB ping pong bi bi kinetics with substrate inhibition demonstrates why CtfAB could be the limiting step in *in vivo* acetone production, where the low binding affinity for acetate and relatively high- affinity for acetyl-CoA creates significant inhibition from acetyl-CoA. As acetoacetyl-CoA is generated from two molecules of acetyl-CoA (by the thiolase), the acetone pathway must function in the presence of significant acetyl-CoA concentrations. As such, future enzyme engineering efforts should focus on reducing the inhibition by acetyl-CoA. Together, the improved enzyme kinetics and structural insights have identified specific properties of CtfABs that need to be altered for improved functionality in the acetone formation pathway.

## Methods

### Cloning and expression of *T. melanesiensis* CtfAB (*T. mel* CtfAB)

WT CtfAB from *T. melanesiensis* (*T. mel*) (Tmel_1136, Tmel_1135; WP_012057350, WP_012057349) was expressed in *E. coli* (Rosetta 2(DE3)pLysS, EMD Millipore) from pRSF backbone, as previously described [9], with the exception that only the CtfA (alpha subunit) was histidine tagged (6 x, N-terminal) and the untagged CtfB (beta subunit) was co-expressed with the CtfA. This is similar to the native expression in *T. melanesiensis* and for *in vivo* thermophilic acetone production [10]. Several mutant *T. mel* CtfABs were also expressed. These were made through site-directed mutagenesis of the WT CtfAB expression plasmid. This was done either through kinase-ligase-DpnI digest (KLD) reactions or through Gibson assembly of PCR amplified and synthetic DNA fragments (gBlocks from Integrated DNA Technologies). All plasmids were fully sequence verified by Sanger sequencing (Azenta Lif Sciences). Mutants generated were CtfB (beta subunit) [E46D, [E46A], [E46S], [I25K], [Q105A], [Q105E], [Q105E, G103A], [F42T, Q44E], [F42T, S45C]; CtfA (alpha subunit) [L25M, F54L, T78L], [P118E]. In addition, CtfAB from *C. beijerinckii* strain DJ033 was expressed in the same manner, as well as the chimeric *T. mel* CtfA – DJ033 CtfB. In total, 14 CtfAB variants were expressed, including WT *T. mel* CtfAB. Plasmids were first constructed and sequence verified in NEB 5-alpha Competent *E. coli* (New England Biolabs), then verified plasmid transformed into Rosetta 2(DE3)pLysS *E. coli* for protein expression. As done previously [9], ZYM-5052 lactose autoinduction media was used to express CtfABs. Briefly, 2 L of ZYM-5052 with 100 mg/L kanamycin and 34 mg/L chloramphenicol (1L media per 2.5 L Fernbach flasks) was inoculated to initial OD_600_ = 0.02 from overnight cultures grown in LB. Autoinduction flasks were grown for 20–24 h until OD_600_ was 10 to 15. Cell pellets were obtained by centrifugation at 6000 x g for 15 min. Pellets were immediately processed to purify CtfAB.

### Enzyme purification

CtfAB were purified from cell pellets, as described previously [9]. Briefly, pellets were resuspended with 5 mL / g-cell-wet-weight in IMAC buffer A (300 mM NaCl, 50 mM NaH_2_PO_4_, 100 mM Na_2_SO_4_, 20 mM imidazole, and 20% glycerol, pH 8.0) then lysed using a French pressure cell. Lysate was heat treated at 65°C for 30 min, then centrifuged at 24,000 x g for 60 min. The supernatant was then 0.22 µm filtered. Cytiva HisTrap HP (5 mL) columns were used for the nickel-histidine affinity chromatography with 5 mL/min flow rates; cell lysate (up to 75 mL) was applied to IMAC buffer A equilibrated columns, then washed for five column volumes (CV) with IMAC buffer A, followed by a gradient to 100% IMAC buffer B (300 mM NaCl, 50 mM NaH_2_PO_4_, 100 mM Na_2_SO_4_, 500 mM imidazole, and 20% glycerol, pH 8.0) over 10 CV, then 100% IMAC buffer B for 5 CV. Five mL fractions were collected. SDS-PAGE was used to screen fractions. Up to 25 mL fractions was pooled and concentrated to 5 mL with 30 kDa cutoff Vivaspin 20 centrifugal concentrators with polyethersulfone filters. This concentrate was loaded onto a HiLoad Superdex 26/600 200 pg size exclusion column, then eluted in SEC Buffer (300 mM NaCl, 50 mM NaH_2_PO_4_, 100 mM Na_2_SO_4_, and 20% glycerol, pH 8.0) at a flowrate of 2 mL/min for 320 min (~2 CV). Four mL of fractions was collected and screened by SDS-PAGE and Native-PAGE. Fractions with good native size purity (based on SEC peak and Native-PAGE) and SDS-PAGE band purity were pooled, concentrated to 10–30 mg/mL (~1–2 mL) in 30 kDA spin concentrators. Glycerol (100%) was added to bring final concentration of 50% glycerol (v/v) and stored at -20°C. Protein concentrations were determined with Pierce Coomass, i.e. (Bradford) Protein Assay kit and Qubit Protein Broad Range Assay kit, per manufacture protocols.

### Native and SDS-PAGE

Samples were separated on NativePAGE Novex 4-16% Bis-Tris gels (Invitrogen) per manufacture instructions for nondetergent samples with the Dark Blue Cathode buffer. Samples were standardized to 1 µg protein per well, and the NativeMark Unstained Protein Standard (Invitrogen) was used. The Fast Coomass, i.e. G-250 Staining protocol, was used. SDS-PAGE gels were run with 4–15% Mini-PROTEAN TGX Stain-free Protein Gels, 15 well (Bio-Rad) per manufacture instructions. SDS-PAGE samples were denatured in 1:1 ratio of sample to 2 x Laemmli Sample Buffer (Bio-Rad) with 5% β-mercaptoethanol at 95°C for 30 min. BenchMark Protein Ladder (Invitrogen) was used.

### Activity assays

CtfAB were assayed by measuring consumption of the magnesium-enolate form of acetoacetyl-CoA on a LAMBDA 365 UV-Vis Spectrophotometer (PerkinElmer) with 310 nm absorbance at 65°C in UV-transparent ultra-micro-cuvettes (BrandTech Scientific). Assays had a final volume of 160 µL and final concentration of 100 mM Tris-HCl, 40 mM MgCl_2_, 5% glycerol, pH 7.5. Forty-eight ng per reaction of CtfAB (diluted in 100 mM Tris-HCl, 5% glycerol, pH 7.5) was used. For the forward reaction (acetoacetate forming direction), acetoacetyl-CoA (sodium salt hydrate, Cayman Chemical item 21219) was supplied between 0.025 and 0.4 mM. Acetyl-CoA (trilithium salt, trihydrate, MP Biomedicals) was supplied between 0 and 1.88 mM for inhibition experiments. Potassium acetate was provided, where it was indicated between 18.75 and 375 mM. Reverse reaction (acetoacetate consuming) assays provided acetyl-CoA between 0.033 and 0.4 mM and potassium acetoacetate between 3.75 and 18.75 mM. Reaction mixtures (90% final volume) were assembled on ice without enzyme; this mixture was heated for 45 sec in the spectrophotometer, following which the reaction was initiated by the addition of enzyme and monitored for 3 min. Non-enzymatic controls were run by the addition of 100 mM Tris-HCl, 5% glycerol, pH 7.5 instead of diluted enzyme. Enzymatic rates were taken by subtracting out non-enzymatic rates of degradation of acetoacetyl-CoA for each set of substrate conditions. Only the initial linear rates after the addition of enzyme were taken for kinetic analysis. Kinetics were fit with Origin Lab non-linear regression tool using the ping-pong bi-bi rate equation for the forward reaction and ping-pong bi-bi without substrate inhibition for the reverse reaction [21] .

*In vitro* acetone formation assays were also used to assess CtfAB. This was done similarly to previous work using recombinantly purified Adc and Thl with detection of acetone on a GC-2014 gas chromatograph (Shimadzu) with a ZB-WAXplus 30 m × 0.53 mm ID capillary column and a flame ionization detection, as described [9].

### Crystallization

Prior to crystallization, purified CtfAB samples were buffer-exchanged from their −20°C storage conditions into crystallization buffer (10 mM MOPS, 5% glycerol, 50 mM Na_2_SO_4_, 50 mM NaCl, 1 mM DTT, pH 7.5). This was accomplished with 30 kDA cutoff Vivaspin 20 concentrators using five sequential 10x dilutions in crystallization buffer and concentration (i.e. 1 mL of −20°C stock with 9 mL crystallization buffer was concentrated to 1 mL, followed by another 9 mL crystallization buffer, and repeated). Crystals of WT CtfAB (CtfAB-WT) were formed in hanging drop trays using 1.5 μL of protein (5–6 mg/mL) in crystallization buffer (10 mM MOPS, 5% glycerol, 50 mM Na_2_SO_4_, 50 mM NaCl, 1 mM DTT, pH 7.5) mixed with 1.5 μL of reservoir solution (100 mM Na acetate pH 4.9–5.5, 6–10% PEG 3350). Protein reservoir mixtures of 1 μL protein:2 μL reservoir and 2 μL protein:1 μL reservoir were also routinely used for crystallization of WT and mutant CtfAB. The active site glutamate (E46) was mutated to serine (E46S) and aspartate (E46D). For data collection, crystals were transferred via cryo-loop to a cryo-protectant solution of reservoir solution supplemented with 25% MPD (Hampton Research) and immediately frozen in liquid nitrogen. Crystals of CtfAB-WT were solved in the space group P4(1)2(1)2 with unit cell dimensions a = b = 131.415, c = 158.901, alpha = beta = gamma = 90. Beta subunit E46S and E46D mutants crystallized under similar crystallization conditions and were all solved in the space group P4(1)2(1)2 with similar unit cell dimensions. A co-crystal of the mutant CtfB F42T/S45C and acetyl-CoA was grown by mixing protein (5–6 mg/mL) with 5–10 mM acetyl-CoA. Co-crystals formed under similar conditions as mutant crystals, and the co-crystal structure was solved in the space group P4(1)2(1)2 with similar unit cell dimensions as the apo-crystals.

#### Data collection and refinement

A low-resolution (3.0 Å) dataset was collected on a home X-ray source, and the structure was solved by molecular replacement in Phenix using a homology model of CtfAB created using the SWISS-PROT Homology Server [29,30]. The asymmetric unit of CtfAB crystals is a heterotetramer composed of two heterodimers of CtfA and CtfB. NCS symmetry between CtfA Chains A and C and CtfB Chains B and D was used as restraints for most of the structure refinement process. High-resolution datasets for WT, mutant, and co-crystals were collected at SERCAT and solved by molecular replacement using the low-resolution structure as the initial model [31]. The CtfAB-WT structure was solved by multiple rounds of reciprocal space and real space refinement in Phenix followed by manual rebuilding in COOT [32]. Solvent molecules, including acetate and water, were built into Fo-Fc difference density contoured at 3.0 Sigma or higher. Difference density for acetate was clearly visible in the active site of CtfAB at the earliest stages of refinement. Apo mutant structures were solved using the CtfAB-WT model. The co-crystal of mutant F42TS45C and acetyl-CoA was solved using the WT apo model. Difference density for the phosphoadenosine portion of acetyl-CoA was clearly visible at the earliest stages of refinement. The pantethione portion of acetyl-CoA remained relatively disordered, with weak electron density and multiple chain breaks in the electron density. Acetyl-CoA ligand difference density was clearly apparent in both heterodimers; however, electron density for acetyl-CoA bound to chain C was more complete than electron density for CoA bound to Chain B, which is missing density for the phosphoadenosine sugar. Difference density for acetate and acetyl-CoA molecules contoured at 3.0 Sigma and calculated from models without ligands added is shown in (Supplementary Figure S4). Data collection and refinement statistics are shown in Table 2.

**Table 2:** Data collection and refinement statistics*

	CtfAB-WT	CtfAB-F42TS45C-CoA	CtfAB-E46D	CtfAB-E46S
PDB code	9CSC	9CTD	9CQ2	9CRY
Wavelength	1.0	1.0	1.0	1.0
Resolution range	36.45–2.001 (2.072–2.001)	41.95–1.9 (1.968–1.9)	47.19–2.2 (2.279–2.2)	41.53–2.6 (2.693–2.6)
Space group	P 41 21 2	P 41 21 2	P 41 21 2	P 41 21 2
Unit cell	131.421 131.421 158.907 90 90 90	130.694 130.694 155.382 90 90 90	131.368 131.368 158.423 90 90 90	131.339 131.339 158.788 90 90 90
Total reflections	1,260,720	1,424,078	1,017,704	596,977
Unique reflections	90,497 (9001)	104,625 (10118)	70,388 (6903)	43,015 (4210)
Multiplicity	13.6 (14.3)	13.4 (13.7)	14.4 (13.1)	13.8 (13.0)
Completeness (%)	96.24 (97.37)	98.75 (96.94)	99.43 (99.14)	99.21 (98.50)
Mean I/sigma(I)	16.23 (3.73)	19.2 (3.2)	14.4 (13.1)	13.49 (2.09)
Wilson B-factor	23.08	17.53	29.2	44.68
R-merge	0.1359 (0.6419)	0.146 (0.620)	0.186 (1.224)	0.2062 (1.223)
R-meas	0.1412 (0.6656)	0.152 (0.644)	0.1929 (1.274)	0.214 (1.271)
CC1/2	0.997 (0.838)	0.990 (0.913)	0.998 (0.785)	0.996 (0.808)
Reflections used in refinement	90,495 (9001)	104,620 (10118)	70,375 (6903)	43,001 (4210)
Reflections used for R-free	1963 (195)	1980 (190)	1990 (194)	1988 (196)
R-work	0.2224 (0.2993)	0.1506 (0.1840)	0.1692 (0.2354)	0.1856 (0.2315)
R-free	0.2856 (0.3692)	0.1766 (0.2327)	0.2193 (0.2816)	0.2325 (0.2983)
# of non-hydrogen atoms	6850	7256	7037	6754
Macromolecules	6392	6440	6419	6391
Ligands	16	269	18	23
Solvent	442	652	600	340
Protein residues	854	854	854	854
RMS(bonds)	0.015	0.012	0.012	0.01
RMS(angles)	1.46	1.27	1.27	1.29
Ramachandran favored (%)	97.28	97.87	97.64	97.4
Ramachandran allowed (%)	2.13	1.65	1.89	2.13
Ramachandran outliers (%)	0.59	0.47	0.47	0.47
Rotamer outliers (%)	2.01	0.57	1.28	1.29
Clashscore	11.42	4.87	4.32	7.24
Average B-factor	26.77	22.09	35.78	45.65
Macromolecules	26.62	20.72	35.42	45.69
Ligands	33.19	47.2	56.67	59.2
Solvent	28.69	29.34	38.95	43.84

* statistics for the highest-resolution shell are shown in parentheses.

## Supplementary material

online supplementary figure 1.

online supplementary figure 2.

online supplementary figure 3.

online supplementary figure 4.

online supplementary figure 5.

## Data Availability

Supporting data are included within the main article and its supplementary files. X-ray crystal structures and associated data are accessible through the Protein Data Bank: PDB IDs: 9CSC (CtfAB-WT) 9CTD (CtfAB-F42TS45C-CoA) 9CQ2 (CtfAB-E46D) 9CRY (CtfAB-E46S) PROTEIN ACCESSION IDs: WP_012057350 CoA transferase subunit A [*Thermosipho melanesiensis*] WP_012057349 3-oxoacid CoA-transferase subunit B [*Thermosipho melanesiensis*]

## Abbreviations

ABE: acetone-butanol-ethanol
Adc: acetoacetate decarboxylase
CV: column volumes
CtfAB: family 1 Coenzyme A transferases
EPPIC: evolutionary protein-protein interface classifier
SCOT: succinyl-CoA:3-oxoacid CoA transferase
SEC: size exclusion chromatography
Thl: thiolase
WT: wild-type
