# Supplementary figures and images for: Structural and kinetic characterization of an acetoacetyl-Coenzyme A: acetate Coenzyme A transferase from the extreme thermophile Thermosipho melanesiensis
Source: Biochem J. 2025 Feb 18;482(4):BCJ20240747. doi: 10.1042/BCJ20240747 (PMC12400318; doi:10.1042/BCJ20240747)

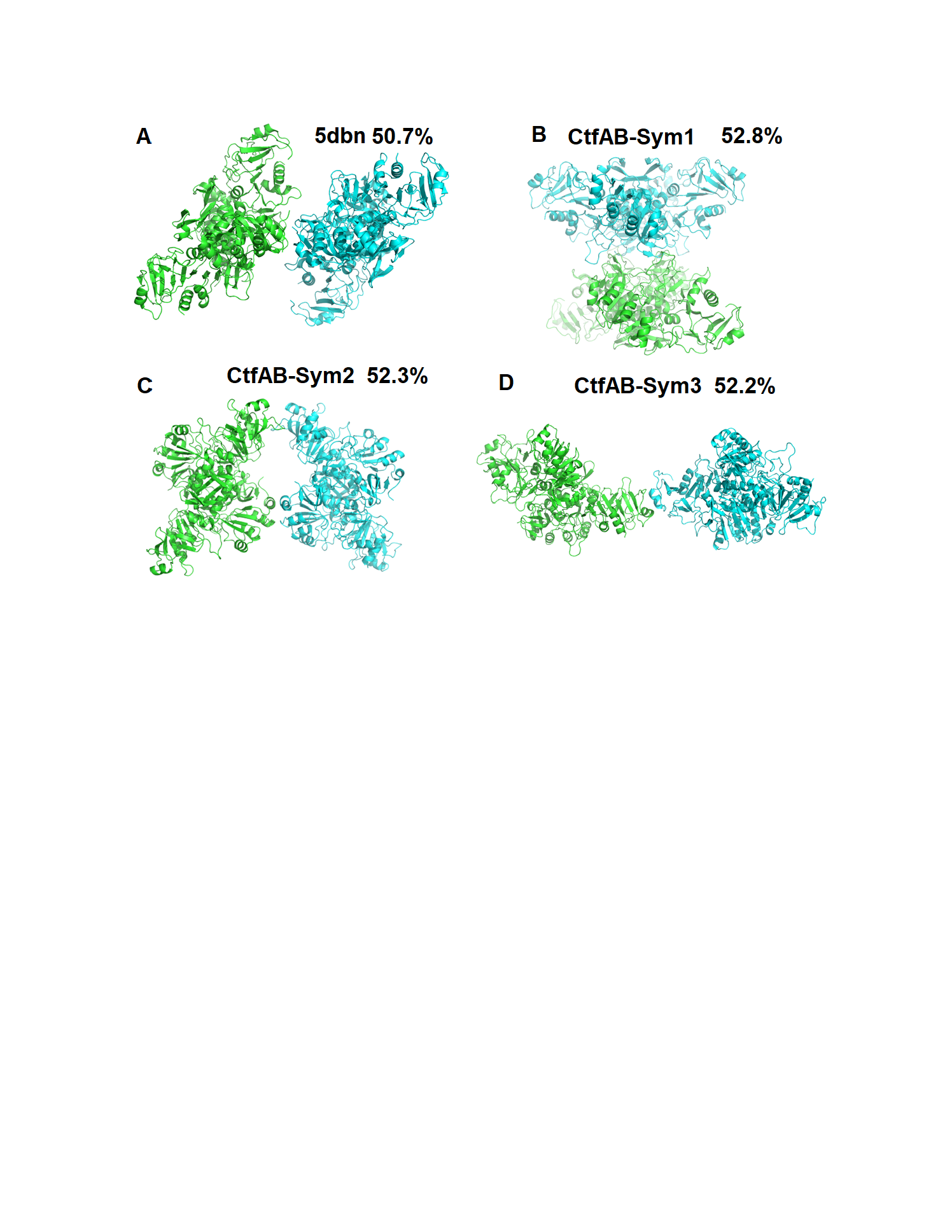

Supplement: online supplementary figure 1. [file bcj-482-4-BCJ20240747-s001.png]

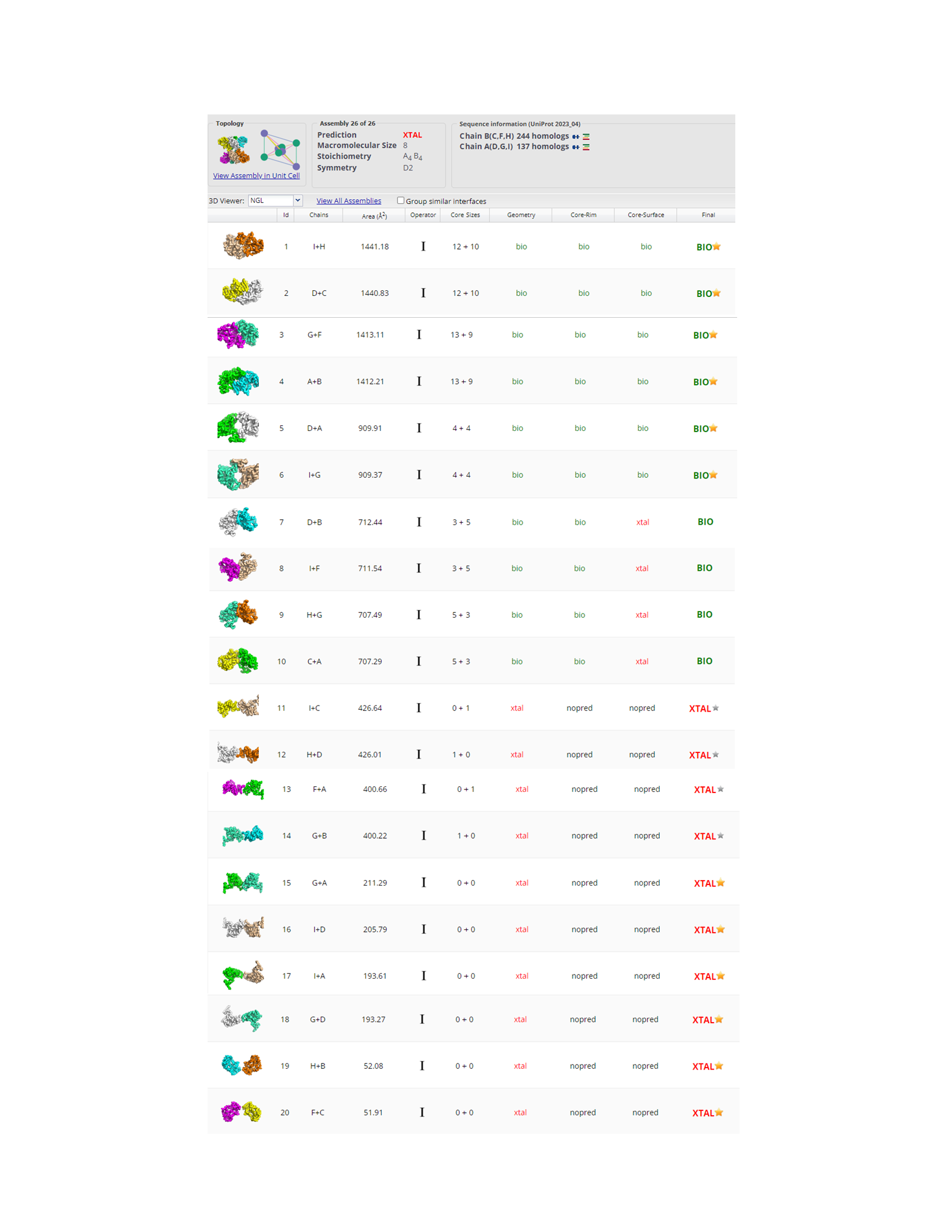

Supplement: online supplementary figure 2. [file bcj-482-4-BCJ20240747-s002.png]

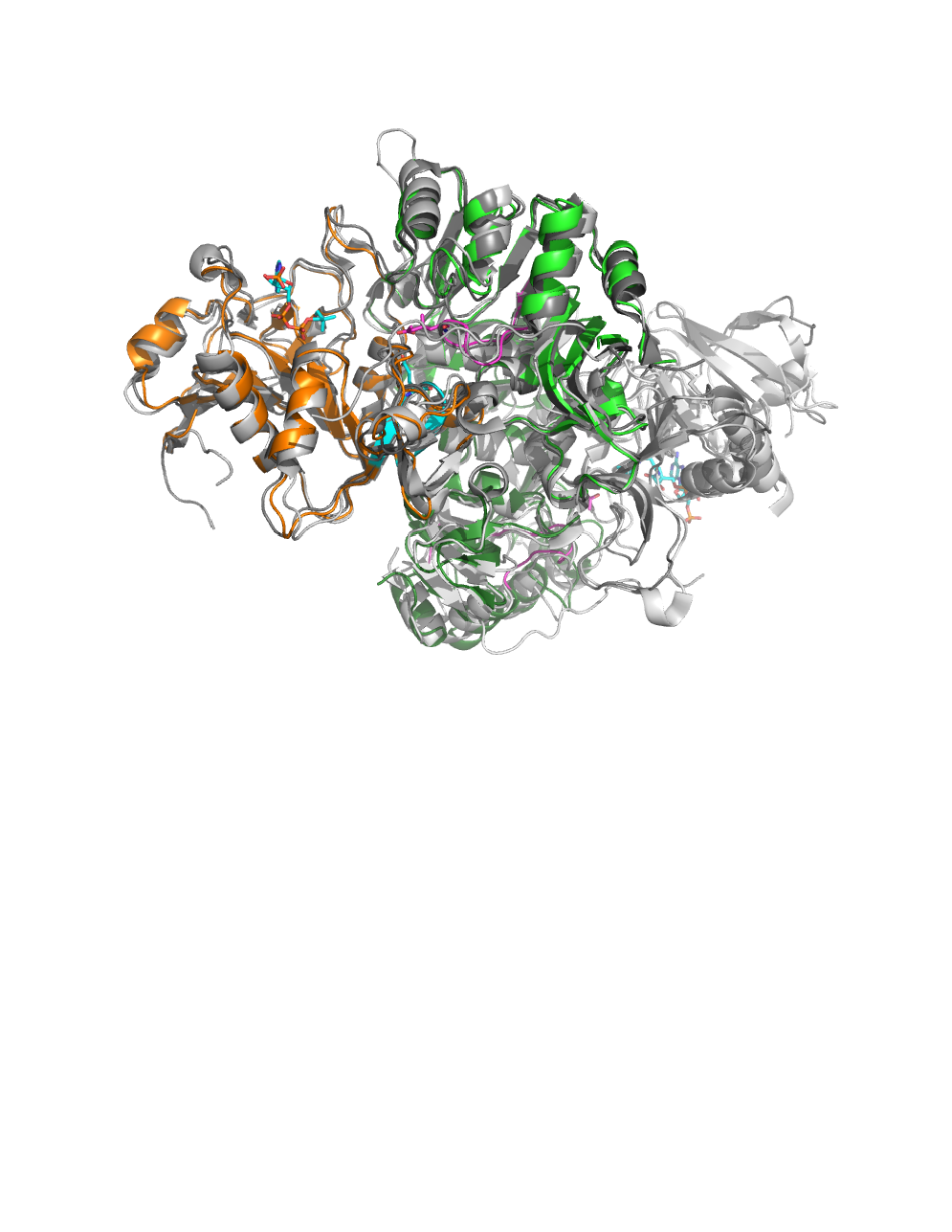

Supplement: online supplementary figure 3. [file bcj-482-4-BCJ20240747-s003.png]

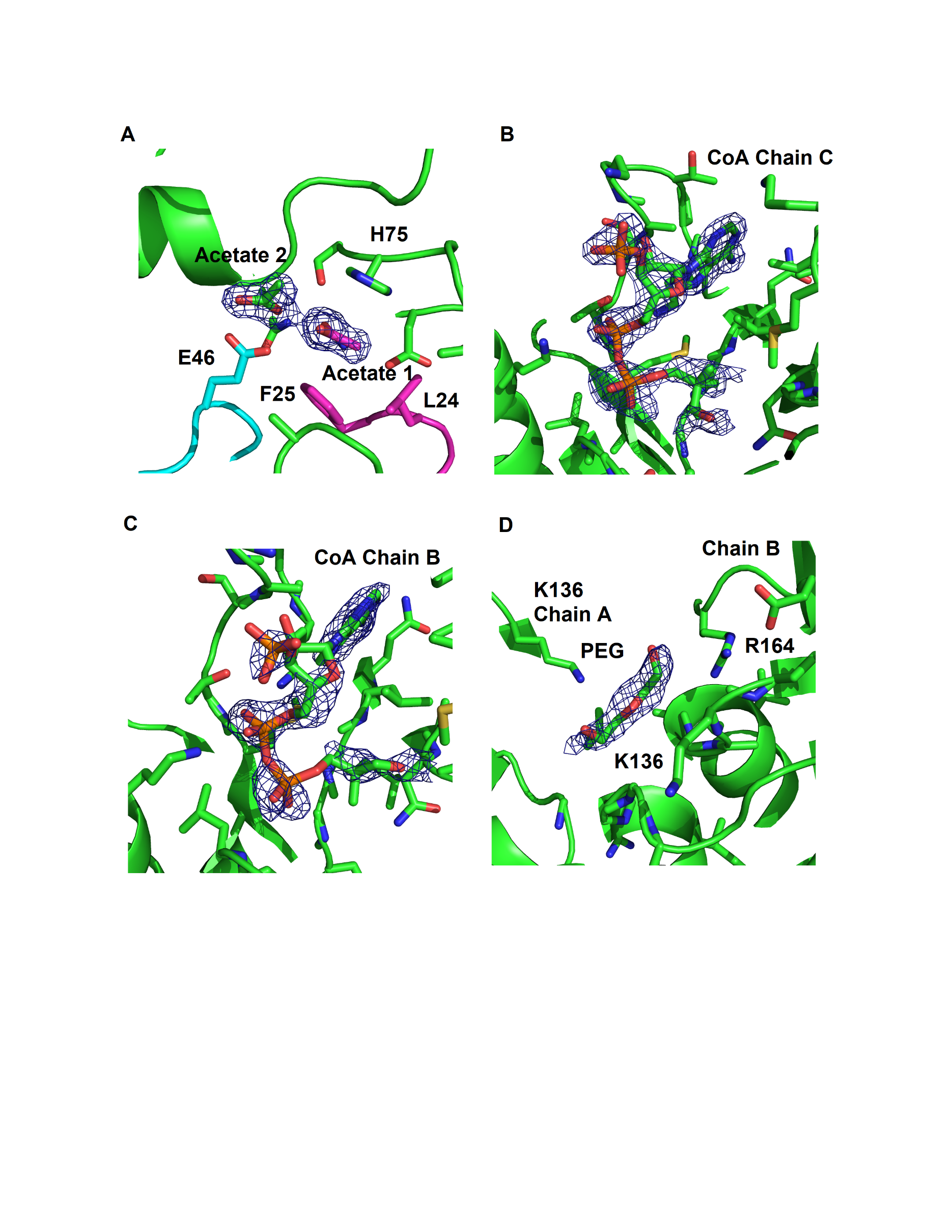

Supplement: online supplementary figure 4. [file bcj-482-4-BCJ20240747-s004.png]

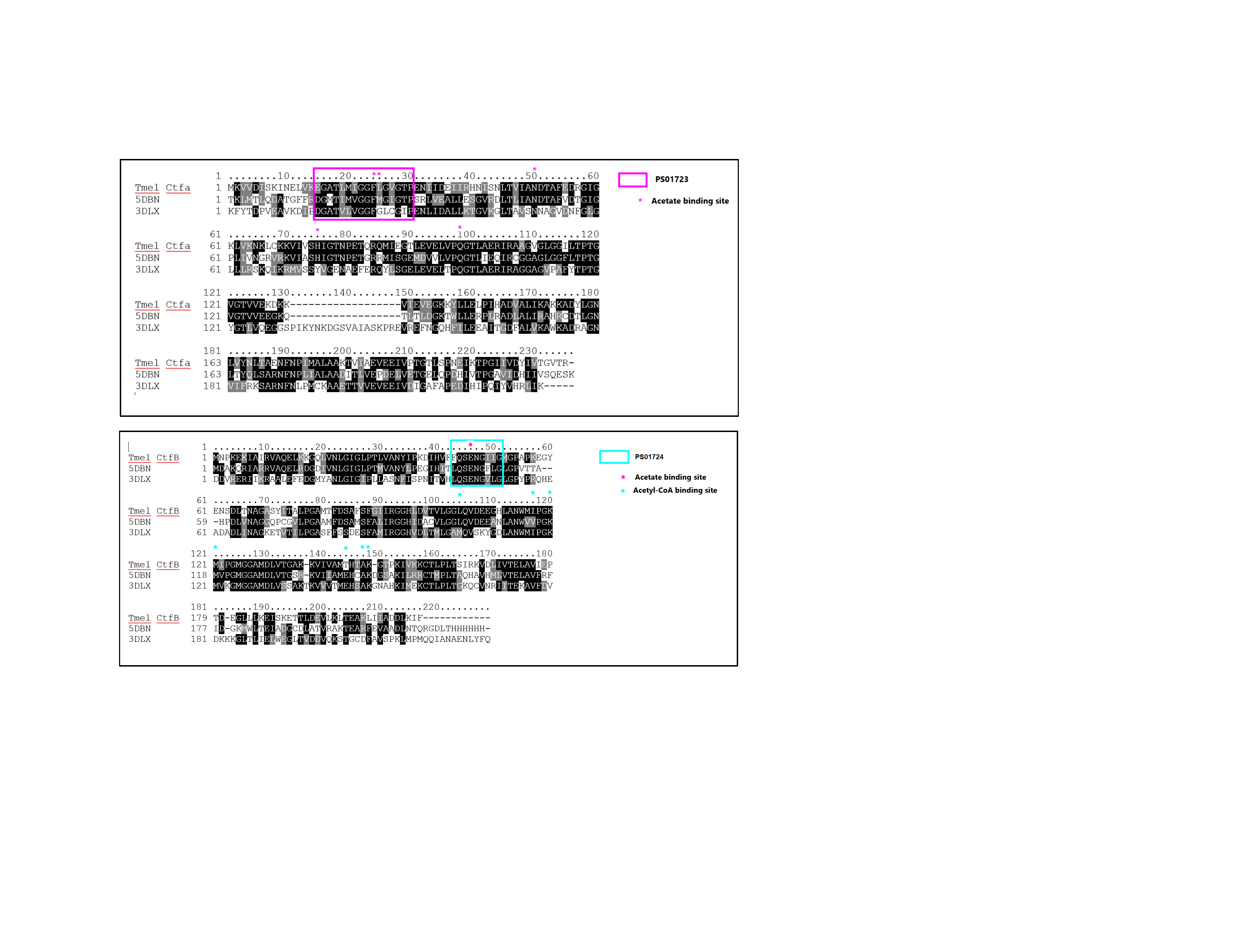

Supplement: online supplementary figure 5. [file bcj-482-4-BCJ20240747-s005.png]
